# Vascular structure and function and their relationship with health-related quality of life in the MARK study

**DOI:** 10.1186/s12872-016-0272-9

**Published:** 2016-05-12

**Authors:** Luis García-Ortiz, José I. Recio-Rodríguez, Sara Mora-Simón, John Guillaumet, Ruth Martí, Cristina Agudo-Conde, Emiliano Rodriguez-Sanchez, Jose A. Maderuelo-Fernandez, Rafel Ramos-Blanes, Manuel A. Gómez-Marcos, the MARK Group

**Affiliations:** Primary Care Research Unit, The Alamedilla Health Center. Castilla and León Health Service (SACyL), Biomedical Research Institute of Salamanca (IBSAL), 37003 Salamanca, Spain; Biomedical and Diagnostic Sciences Department, University of Salamanca, Salamanca, Spain; Department of Medicine, University of Salamanca, Salamanca, Spain; Basic Psychology, Psychobiology and Behavioral Sciences Methodology Department, University of Salamanca, Salamanca, Spain; San Agustín Health Center, Illes Balears Health Service (IBSALUT), Palma de Mallorca, Spain; Research Unit Family Medicine, Girona. Jordi Gol Institute for Primary Care Research (IDIAP Jordi Gol), Catalonia, Spain; Translab Research Group. Medical Sciences Department, School of Medicine, University of Girona, Girona, Spain; Girona Biomedical Research Institute (IDIBGI), Dr. Trueta University Hospital, Girona, Spain

**Keywords:** Health-related quality of life, Arterial stiffness, Ankle-brachial index, Brachial ankle pulse wave velocity, Cardio-ankle vascular index, Augmentation index

## Abstract

**Background:**

There is limited evidence concerning the relationship between vascular disease and health-related quality of life (HRQL). We investigated the relationship between vascular structure and function with health-related quality of life in a population with intermediate cardiovascular risk.

**Methods:**

This study analyzed 303 subjects with ankle-brachial index (ABI) values ranging from 0.9 to 1.4 who were included in the MARK study (age 35 to 74 years; mean:60.5 ± 8.5), of which 50.2 % were women. Measurements included: ABI, brachial-ankle pulse wave velocity (ba-PWV), and cardio-ankle vascular index (CAVI), all measured using the VaSera device. The central augmentation index was adjusted to 75 lpm (AIx_75) using the Mobil-O-Graph device. HRQL was assessed by the Spanish version of the SF-12, version2. The highest obtained CAVI and ba-PWV values and the lowest ABI values were considered for the study.

**Results:**

The cohort was composed of21 % smokers, 76 % hypertensive patients, and 24 % diabetic patients. The ABI mean was 1.09 ± 0.07,the ba-PWV mean was 14.64 ± 2.55 m/s with a 12.9 % of subjects higher than 17.5 m/s, AIx_75 26.46 ± 14.05, and CAVI 8.61 ± 1.08 with a 36.6 % of subjects higher than 9. Men scored higher than women in the HRQL measurements for physical (PSC-12; 49.9 vs. 46.9, *p* = 0.004) and mental (MSC-12) domains (51.2 vs. 47.7, *p* = 0.003). Age was positively correlated with CAVI (*r* = 0.547), ba-PWV (*r* = 0.469), AIx_75 (*r* = 0.255, *p* < 0.01), and the MSC-12 (*r* = 0.147, *p* < 0.05), but not the PSC-12. In the adjusted multiple linear regression analysis, the positive association of ABI and CAVI with the PSC-12 was maintained.

**Conclusions:**

The ABI in the normal range has a positive association with the PSC-12 of HRQL evaluated with the SF-12. The CAVI also showed a positive association with the PSC-12 of HRQL.

**Trial Registration:**

ClinicalTrials.gov Identifier: NCT01428934.

**Electronic supplementary material:**

The online version of this article (doi:10.1186/s12872-016-0272-9) contains supplementary material, which is available to authorized users.

## Background

Health outcomes reported by patients are becoming more important in research, clinical practice, and health planning [1, 2]. Self-perception of health status and health-related quality of life (HRQL) provide information that complements traditional health indicators based on morbidity and mortality [3]. HRQL is an important outcome in clinical trials, population health assessments, clinical improvement, and documenting for purchasers quality of care. In addition to the more objective clinical measures, many patients consider HRQL equally important. HRQL is conceptualized as a patient's perceptions of the impact of disease and treatment on functioning in a variety of dimensions, including physical, mental, and social domains [4, 5]. One of the most commonly used instruments to measure HRQL is the SF-36 Questionnaire or its SF-12 version [3, 6], that reduces the workload of health workers, the workload for patients, and the time to complete the questionnaire.

The assessment of vascular structure and function with different devices allows for the detection of the early stages of atherosclerosis and the degree of arterial stiffness. Fowkes et al. conducted a meta-analysis of 16 population cohort studies that included 480.325 person years of follow up, and found that the ankle–brachial index (ABI) showed an inverse linear relationship between subclinical peripheral arterial disease (PAD) and cardiovascular disease, even at ABI values between 0.91 and 1.00 [7]. The authors also found that the risk of death for different levels of ABI, compared with a reference ABI score of 1.11 to 1.20, formed a reverse J-shaped curve. For levels of ABI below 1.11, the hazard ratios (HRs) increased with decreasing ABI, and for ABI >1.40 the HRs also increased, but this was not the case for ABI scores between 1.11 and 1.40 [7]. However, little is known about the relationship between ABI and activities of daily living functioning at the population level. In some subgroups of subjects with high cardiovascular risk, PAD and severe renal impairment [8–10], there was a positive association between ABI and HRQL, including for patients whose ABI was in the range 0.9 to 1.3. A worse quality of life was also found in subjects with ABI > 1.4 [11]. The subgroup of patients with intermediate cardiovascular risk is the group in which the highest number of cardiovascular events occur, and it is known the association of these with a worse HRQL [12, 13]. However, the potential influence of ABI on HRQL, when considering ABI as a continuous variable, in individuals with intermediate cardiovascular risk and ABI in the normal range [14] has not been analyzed. Knowing this relationship may lead to improvements in a multidimensional therapeutic approach for this very large subjects group at risk for a cardiovascular event.

Vascular function, as evaluated by pulse wave velocity (PWV) [15, 16], has been correlated with morbidity and mortality both in patients with cardiovascular disease and in healthy individuals. The cardio-ankle vascular index (CAVI) is a parameter [17] of the overall stiffness of the artery from the aorta's origin to the ankle. It can be used to estimate the risk of atherosclerosis [18]. The relationship between vascular function and HRQL has been little studied, and only in some population subgroups. Bruner et al. [19] found a negative relationship between the PWV and the physical component of quality of life, as assessed by the SF-36 in an English-speaking general population. Likewise, Crilly et al. [20] showed a positive association between the Stanford disability index questionnaire and the augmentation index (AIx) in patients with rheumatoid arthritis who were free of overt arterial disease. However, we are unaware of any study focusing on the relationship between CAVI and HRQL. These vascular function measures are newly developed and evaluate the early stages of atherosclerosis, and have not been studied in subjects with intermediate cardiovascular risk. An important implication is whether or not subclinical vascular lesions detected in the early stages of atherosclerotic process affects quality of life, in order to inform the planning of a new therapeutic approach once the alteration of vascular function has been detected. Therefore, the aim of this study is to analyze the relationship of vascular structure and function as assessed by ABI, CAVI, PWV and AIx with HRQL as assessed by the SF-12 questionnaire in a population with intermediate cardiovascular risk.

## Methods

### Study design

The MARK study [21] is a cross-sectional study to evaluate if ABI, CAVI, postprandial glucose, glycosylated hemoglobin, self-measured blood pressure, and comorbid conditions are independently associated with incidences of vascular events, and whether they can improve the predictive capacity of current risk equations in an intermediate risk population. The second step will occur at five and 10 years follow-up to evaluate cardiovascular morbidity and mortality. The current study analyzed 303 subjects who were included and evaluated the HRQL.

### Study population

The population consisted of individuals between 35 to 74 years of age who had intermediate cardiovascular risk, which was defined as coronary risk between 5 and 15 % at 10 years according to the Adaptation of the Framingham-Wilson coronary risk equation (REGICOR) [22] or vascular mortality risk between 1 % and 5 % at 10 years according to the SCORE equation [23] or moderate risk according to the 2013 European Society of Hypertension guidelines for the management of arterial hypertension [24]. Exclusion criteria included terminal illness, institutionalization at the appointment time, or a personal history of atherosclerotic disease. Sample selection was performed with a random sampling of the study population that matched the inclusion criteria. Recruitment and data collection were performed from July 2011 to June 2013. The quality of life questionnaire was performed in the last 314 of the 500 subjects recruited in Salamanca. Eleven patients were excluded because they did not have CAVI values or had an ABI <0.9 or > 1.4. Thus, 303 subjects were analyzed.

A sample-size calculation indicated that the 303 patients included in the study constituted a sufficient sample to detect a five point difference in the standardized physical or mental component of the SF-12 between the three categories of CAVI, which is considered a clinically important difference (CID) according Parker SL et al. [25]. We considered a common standard deviation of 10 points, with a level of significance of 95 % and a power of 80 % in a two-sided test. Parker SL et al. [25] found a CID of 4.7 and 8.1 points in the SF-12 for the MCS-12 and PCS-12, respectively. We used the EPIDAT 4.0 software to perform this calculation.

The study was approved by an independent ethics committee from the Salamanca health area (Spain), and all participants gave written informed consent according to the general recommendations of the Helsinki Declaration [26].

### Measurements

A detailed description has been published elsewhere regarding how the clinical data, drugs therapy, anthropometric measurements, and analytical parameters were obtained from patients [18].

### Health-related quality of life (HRQL)

HRQL was assessed with the Spanish version of the SF-12v.2, which has been validated [3, 6]. The SF-12 is a shorter version of the SF-36 questionnaire [27], and includes 12 items, with 3 to 5 response categories placed on a Likert scale. The SF-12 questionnaire is self-administered and was developed to measure eight dimensions of HRQL: Physical Functioning, Role Physical, Body Pain and General Health scales, Vitality, Role Emotional, Social Functioning, and Mental Health. These eight dimensions can be aggregated into two summary measures: a physical component summary (PCS-12) and a mental component summary (MCS-12). To estimate the summary components of the SF-12 (i.e., PCS-12 and MCS-12), we calculated the algebraic sum of the standardized scores of the eight dimensions (z scores) weighted by weights (see supplementary material). The Physical and Mental Health Composite Scores (PCS-12 & MCS-12) are computed using the scores of the 12 questions and range from 0 to 100, where a zero indicates the lowest level of health measured by the scales and 100 indicates the highest level of health Additional file 1: Table S1 [28]. The values are standardized to a United States norm with a mean of 50 and a standard deviation of 10. Thus, the SF-12 summaries compare the scores for each study participant against the mean score in the population. A higher score in the PCS-12 or the MCS-12 corresponds to better health status. The two standardized summary scores provide a concise approximation of the physical and mental components of HRQL [28].

### Vascular assessment

The ankle/brachial index (ABI), cardio ankle vascular index (CAVI), and brachial ankle pulse wave velocity (ba-PWV) were measured using a VaSera VS-1500® device (Fukuda Denshi). The ABI was calculated automatically for each foot by dividing the systolic blood pressure (SBP) in the ankle by the SBP in the arm. An ABI less than 0.9 or greater than 1.4 was considered abnormal [14]; these patients were excluded. CAVI integrates the cardiovascular elasticity derived from the aorta to the ankle pulse velocity through an oscillometric method. Itis a good measure of vascular stiffness, and it does not depend on blood pressure (BP) [29]. The CAVI values were automatically calculated by substituting the stiffness parameter β in the following equation to detect the vascular elasticity and the heart-ankle PWV: Stiffness parameter β = 2ρ × 1/(Ps –Pd) × ln (Ps/Pd) × ha-PWV2, where ρ is the blood density, Ps and Pd are SBP and diastolic blood pressure (DBP) in mmHg, respectively, and the ba-PWV is measured between the aortic valve and the ankle. The average coefficient of the variation of the CAVI is less than 5 %, which is small enough for clinical use and confirms that the CAVI has favorable reproducibility [30]. The CAVI was measured at rest and was considered normal when CAVI < 8, borderline if CAVI ≥8 or < 9, or abnormal with suspicion of subclinical atherosclerosis if CAVI ≥9. The ba-PWV was estimated using the following equation: ba-PWV = (0.5934 × Height (cm) + 14.4724)/tba, where tba is the time difference between the time when pulse waves were transmitted to the brachium and the time when these same waves were transmitted to the ankle [31]. A ba-PWV ≥17.5 m/s was considered abnormal [32]. We used the highest CAVI and ba-PWV and the lowest ABI obtained from right or left measurements.

The Augmentation Index (AIx) was estimated using an oscillometric Mobil-O-Graph (Stolberg, Germany) [33]. The measurements of central SBP (cSBP) and peripheral SBP (pSBP) were taken in the dominant arm. Arm circumference was measured and recorded to allow for the correct choice of cuff size (two sizes available: 24–34 and 32–42 cm). With a conventional cuff, the determination of the cSBP is based on an oscillometric BP measurement and uses the pulse waves assessed at the brachial artery. After estimating the pBP, the cuff instantly re-inflates and recordings of the cSBP are performed at DBP levels for 10 s [34]. From the morphology of the aortic wave, the AIx was estimated using the following formula: increase in central pressure × 100/pulse pressure. The values were adjusted to a heart rate of 75 (AIx_75) by the Mobil-O-Graph device [33].

### Lifestyle health behavior variables

#### Smoking

Smoking history was assessed through questions about the participant’s smoking status (smoker/non-smoker). We considered smokers to be subjects who currently smoke or who stopped smoking less than one year ago.

#### Alcohol

Alcohol consumption was assessed through a structured questionnaire and was expressed in grams per week.

#### Physical activity

Leisure time physical activity (LTPA) practices were collected with the Minnesota LTPA questionnaire, which has been validated for Spanish men and women [35, 36]. The questionnaire was administered by trained interviewers and collected detailed information about physical activity (PA) in the preceding year, the number of times this activity was performed, and the average duration of each activity on each occasion. Each PA has an intensity code based on the ratio between the metabolic rate during PA practice and the basal metabolic rate. We assumed that 1 MET (basal metabolic equivalent) approximately corresponds to 1 kcal/(kg x hour) of energy expenditure, which allowed us to calculate the total energy expenditure in leisure time PA in kilocalories per week. Consumption (MET-min) was estimated over 14 days by multiplying the MET in physical activity by the duration (in minutes) and cumulative frequency in the month prior to the interview.

#### Diet assessment

Diet was evaluated by the Diet Quality Index (DQI) [37]. The DQI includes 18 food groups divided into three categories of consumption, with the exception of alcoholic beverage consumption. Daily intake of one portion of foods in the first food category is scored with two points; lower and higher intake portions are scored with one and three points, respectively. Daily consumption of one alcoholic drink (i.e., 1 bottle of beer, 1 glass of wine, or 1 cup of liquor equivalent to approximately 12 g of alcohol) is scored with three points; lower and higher intakes are scored 1. Consumption of foods considered detrimental (i.e., in the second food group category) is scored with two points if frequency occurs between four and six times per week; greater and lower frequent consumption patterns are scored at one and three points, respectively. High consumption (i.e., four or more times per week) of food items considered beneficial (i.e., in the third food group category) are scored with three points, whereas intake of two to three times per week and less than twice a week are scored with two and one points, respectively. All food item scores are added up. The total possible score ranges from 18 to 54.

### Others measurements

#### Anthropometric measurements

Body mass index (BMI) was calculated as weight (kg) divided by height squared (m^2^). A value >30 kg/m^2^ was defined as obese.

#### Office blood pressure

Office BP was calculated as the average of the last two of three measurements of SBP and DBP, made with a validated sphygmomanometer (OMRON Model M10-IT). Measurements were made on the dominant arm while participants were in a seated position after at least five minutes, with a cuff of appropriate size as determined by measurement of the upper arm circumference and following the recommendations of the European Society of Hypertension [38]. Mean arterial pressure (MAP) was calculated as the sum of the SBP and twice the DBP, divided by three.

#### Laboratory determinations

Venous blood sampling was performed between 08:00 and 09:00, after the individuals had fasted and abstained from smoking, alcohol, and caffeinated beverages for the previous 12 h. Fasting plasma glucose, hemoglobin A1c (HbA1c), creatinine, total cholesterol, as well as triglyceride and high-density lipoprotein (HDL) cholesterol concentrations were measured using standard enzymatic automated methods. Low-density lipoprotein (LDL) cholesterol was estimated by the Friedewald equation. Atherogenic index was estimated by total cholesterol/HDL-cholesterol. All assessments were made within a period of 10 days.

### Statistical analysis

Continuous variables were expressed as the mean ± standard deviation for normally distributed continuous data. The median (interquartile range: IQR) was used for asymmetrically-distributed continuous data, and the frequency distribution was used for categorical data. Statistical normality was tested using the Kolmogorov–Smirnov test. The student’s t-test was used to contrast HRQL dimensions by gender. Pearson’s correlation was performed to analyze relationships between quantitative variables. We performed four multiple linear regression analyses using the Multivariate General Linear Model (GLM). The four independent variables including were the ABI, CAVI, ba-PWV, and AIx_75. The eight SF-12 dimensions and PCS-12 and MCS-12 were the dependent variables. The first model was an unadjusted regression, and the second model adjusted for current smoking status, alcohol consumption (g/week), physical activity (METs-min 14 days), nutrition (DQI), MAP, atherogenic index, HbA1c, as well as antihypertensive and lipid-lowering drugs. Comparisons of the physical and mental component between the three categories of CAVI were performed using the multivariate analysis of variance (MANOVA), adjusting for the same variables of multiple linear regressions (GLM) and estimating marginal means. The differences between groups were assessed using the Fisher's Least Significant Difference (LSD) post hoc test. The data were analyzed using the Statistical Package for the Social Sciences version 20.0 (SPSS, Chicago, IL, USA). A value of *p* < .05 was considered statistically significant.

## Results

Table 1 shows the general characteristics of the sample. Of the 303 patients, the mean age was 60.5 ± 8.5 years; 50.5 % were women, 21 % were smokers, 76 % were hypertensive, and 24 % were diabetic. The ABI mean was 1.09 ± 0.07, the ba-PWV mean was 14.64 ± 2.55 m/s with a 12.9 % of patients higher than 17.5 m/s, theAIx_75 mean was of 26.46 ± 14.05 and the CAVI mean was 8.61 ± 1.08 with a36.6 % higher than 9.

**Table 1:** Demographic, clinical and biological characteristics of the sample

		Mean/Median/Num	SD/IQR/(%).
Age		60.48	8.52
Gender (women) n (%)		153	(50.5)
Smoker n (%)		64	(21.1)
Alcohol drinking (g/week)		30	0–90
METs-min/14 days		3536.56	3395.87
Diet quality index		31.30	2.73
Systolic blood pressure (mmHg)		132.96	15.80
Diastolic blood pressure (mmHg)		80.13	10.26
Mean blood pressure (mmHg)		97.74	10.91
Heartrate (bpm)		64.60	10.20
Total Cholesterol (mg/dl)		220.60	38.40
LDL Cholesterol (mg/dl)		139.09	34.26
HDL-Cholesterol (mg/dl)		56.43	14.20
Triglycerides (mg/dl)		106	78–144
Atherogenic index		4.13	1.19
Glycaemia (mg/dl)		88	91–98
HbA1c %		5.6	5.4–6.0
Creatinine (mg/dl)		0.85	0.18
ABI		1.09	0.07
ba-PWV (m/s)		14.64	2.55
ba-PWV > 17.5 m/s *n* (%)		39	(12.9)
CAVI		8.61	1.08
CAVI categories. *n* (%)	<8	90	(29.7)
	8–9	102	(33.7)
	>9	111	(36.6)
AIx_75 %		26.46	14.05
Hypertensive patients *n* (%)		230	(75.9)
Diabetic patients *n* (%)		73	(24.1)
Obesity patients *n* (%)		81	(26.7)
Antihypertensive drugs *n* (%)		146	(48.2)
Antidiabetic drugs *n* (%)		41	(13.5)
Lipid lowering drugs *n* (%)		107	(35.3)

*METs* Metabolic equivalent, Atherogenic index: Total Cholesterol/HDL-Cholesterol, *ABI* Ankle brachial index, *ba-PWV* Brachial-Ankle Pulse Wave Velocity. *CAVI* Cardio Ankle Vascular index, *AIx_75* Augmentation Index adjusted 75 bpm, *SD* Standard Deviation, *IQR* Interquartile Range

The mean HRQL dimensions for the sample (Table 2) are only above the middle (50) for physical function, role physical, and vitality. The scores for the other subscales are close to 50except for general health, which is the lowest (39.92 ± 8.90). Men had higher scores on all dimensions except general health and vitality. They also had higher scores on the PCS-12 (49.9 vs. 46.9, *p* = 0.004) and MCS-12 (51.2 vs. 47.7, *p* = 0.003).

**Table 2:** Health related quality of life dimensions (SF-12) by sex

	Global		Women		Men		p
	Mean	SD	Mean	SD	Mean	SD	
Standardized physical function	50.80 ± 8.92		49.17 ± 9.49		52.46 ± 8.00		0.001
Standardized role physical	50.96 ± 9.13		49.41 ± 9.66		52.54 ± 8.29		0.003
Standardized bodily pain	49.88 ± 11.58		46.72 ± 12.63		53.10 ± 9.40		<0.001
Standardized general health	39.92 ± 8.90		39.24 ± 9.18		40.60 ± 8.59		0.185
Standardized vitality	50.90 ± 11.13		49.85 ± 10.99		51.98 ± 11.20		0.097
Standardized social functioning	49.90 ± 9.72		48.38 ± 10.28		51.45 ± 8.89		0.006
Standardized role emotional	48.94 ± 9.68		47.31 ± 9.95		50.60 ± 9.13		0.003
Standardized mental health	49.67 ± 10.42		47.01 ± 10.04		52.39 ± 10.12		<0.001
Standardized physical component	48.42 ± 9.32		46.90 ± 10.50		49.97 ± 7.66		0.004
Standardized mental component	49.44 ± 10.45		47.66 ± 10.78		51.24 ± 9.82		0.003

*P* values estimated by T-Student test. *SD* Standard Deviation

Age was positively correlated with CAVI (*r* = 0.547), ba-PWV (*r* = 0.469), and AIx_75 (*r* = 0.255), (*p* < 0.01). The relationship was similar with the general health (*r* = 0.117), social functioning (*r* = 0.147), mental health (*r* = 0.148), and MCS-12 (r = 0.147), (*p* < 0.05). However, the relationship did not reach statistical significance for ABI (*r* = 0.051) or PCS-12 (*r* = 0.013).

The multiple linear regressions (Table 3 and Additional file 2: Table S2) considered the dimensions of quality of life as dependent variables. The unadjusted model 1 shows a positive relationship between physical function, role physical, bodily pain dimensions, and the PCS-12 with ABI and CAVI. In addition, CAVI and ba-PWV also have a positive association with the mental health dimension. In the second adjusted model, the positive association of ABI and CAVI with physical function and the PCS-12 was maintained, and a negative association between ABI with the MCS-12 was found (*p* = 0.048).

**Table 3:** Multiple linear regression analysis of vascular structure and function parameters and health-related quality of life

	Model 1: Unadjusted			Model2: Fully adjusted		
	B	CI 95 %	p	B	CI 95 %	p
ABI						
Standardized physical component	24.46	10.40 to 38.52	0.001	23.90	8.24 to 39.55	0.003
Standardized mental component	1.53	−14.55 to 17.61	0.852	−17.43	−34.67 to −0.19	0.048
CAVI						
Standardized physical component	1.50	0.54 to 2.46	0.002	1.59	0.32 to 2.85	0.014
Standardized mental component	0.81	−0.28 to 1.90	0.145	−0.72	−2.11 to 0.67	0.310
PWV						
Standardized physical component	0.19	−0.22 to 0.61	0.361	0.01	−0.53 to 0.52	0.993
Standardized mental component	0.29	−0.18 to 0.75	0.226	−0.08	−0.65 to 0.50	0.791
AIx_75						
Standardized physical component	−0.08	−0.15 to 0.01	0.039	−0.08	−0.17 to 0.01	0.059
Standardized mental component	0.02	−0.06 to 0.11	0.625	0.06	−0.03 to 0.16	0.183

Multiple linear regression analysis by Multivariate General lineal model (GLM). Dependent variables: Standardized Physical Component and Standardized Mental Component

Independent Variables: Ankle brachial index (ABI), Cardio Ankle Vascular index (CAVI), Brachial-Ankle Pulse Wave Velocity (ba-PWV) and Augmentation index (AIx_75)

Model 1: Unadjusted; Model 2: Adjusted for age, gender, current smoker, alcohol consumption in gr/week physical exercise (METs-min 14 days), diet quality index, mean blood pressure, atherogenic index (Total Cholesterol/HDL-Cholesterol), HbA1c and antihypertensive and lipid-lowering drugs

**Fig. 1:**
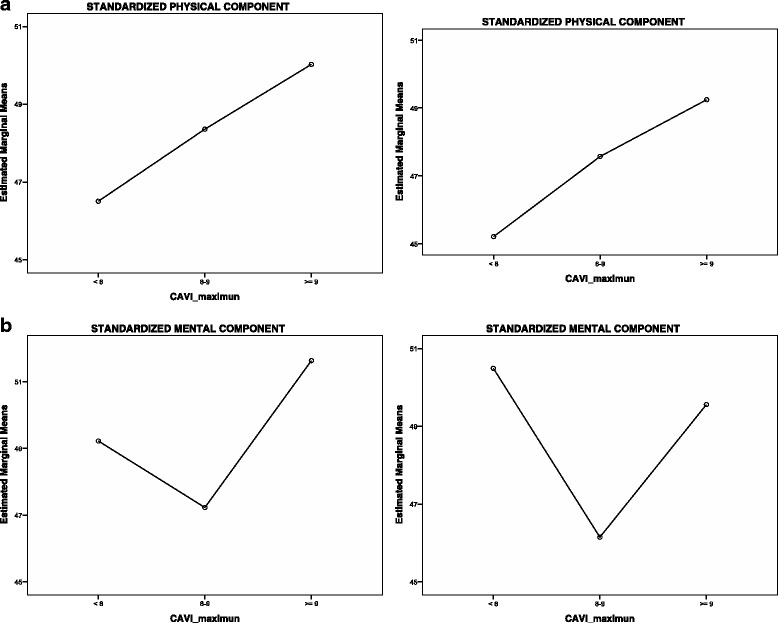
Marginal means of standardized physical (PCS-12) and mental (MCS-12) components by CAVI categories. *P* < 0.05 by Multivariate General Lineal Model (MANOVA) using LSD post hoc test. Unadjusted: PCS-12: CAVI < 8 with > 9. MCS-12: CAVI 8–9 with > 9. Adjusted: PCS-12: CAVI < 8 with > 9. MCS-12: CAVI < 8 with 8–9 and 8-9 with > 9. Adjusted for age, gender, current smoker, alcohol consumption in gr/week, physical activity (METs-min 14 days), diet quality index, mean blood pressure, atherogenic index (Total Cholesterol/HDL-Cholesterol), HbA1c and antihypertensive and lipid-lowering drugs. CAVI: Cardio Ankle Vascular index, SE: Standard error

Figure 1 and Table 4 shows data from the PCS-12 and MCS-12 in relation to the three categories of CAVI. The data highlight a positive linear relationship between the PCS-12 and CAVI, as well as increased marginal PCS-12 means as CAVI increases. This is not the case for the MCS-12, either in the unadjusted or adjusted model.

**Table 4:** Marginal means of standardized physical and mental components by CAVI categories

		Unadjusted	Fully adjusted
	CAVI	Mean ± SE	Mean ± SE
Standardized Physical Component	<8	46.51 ± 0.97*	45.21 ± 1.27*
	8–9	48.36 ± 0.91	47.57 ± 1.06
	> = 9	50.03 ± 0.88*	49.24 ± 1.08*
Standardized Mental Component	<8	49.22 ± 1.09	50.49 ± 1.38
	8-9	47.23 ± 1.02*	45.21 ± 1.27*
	> = 9	51.64 ± 0.98*	47.57 ± 1.06*

*P by Multivariate General Lineal Model (MANOVA) using LSD post hoc test. Unadjusted: Physical Component, CAVI < 8 with 8–9, *p* = 0.167; CAVI < 8 with > 9, *p* = 0.008; CAVI 8–9 with > 9, *p* = 0.190. Mental Component: CAVI < 8 with 8–9, *p* = 0.183; CAVI < 8 with > 9, *p* = 0.101; CAVI 8–9 with > 9, *p* = 0.002. Adjusted: Physical Component, CAVI < 8 with 8–9, *p* = 0.123; CAVI < 8 with > 9, *p* = 0.017; CAVI 8–9 with > 9, *p* = 0.226. Mental Component: CAVI < 8 with 8–9, *p* = 0.009; CAVI < 8 with > 9, *p* = 0.608; CAVI 8–9 with > 9, *p* = 0.023

Adjusted for age, gender, current smoker, alcohol consumption in g/week, physical exercise (METs-min 14 days), diet quality index, mean blood pressure, atherogenic index (Total Cholesterol/HDL-Cholesterol), HbA1c and antihypertensive and lipid-lowering drugs, *CAVI* Cardio Ankle Vascular index, *SE* Standard error.

## Discussion

In a sample of subjects with intermediate cardiovascular risk and an ABI score between 0.9 and 1.4, we found a positive association between ABI and the physical dimensions of HRQL; the higher ABI, the better the patient’s quality of life in the physical dimension. T his positive association was maintained with physical function dimension, the PCS-12, and the MCS-12after adjusting for potential confounders. We found no relationship, after adjustments, between the ba-PWV and any of the dimensions of quality of life. However, we found an inverse unadjusted relationship between the physical function dimension and the AIx_75, and this relationship achieved borderline significance after adjusting (*p* = 0.052). This finding suggests that higher AIx_75 would be associated with worse quality of life in the physical function dimension. CAVI has a positive relationship with HRQL in the physical dimensions. This is true even after adjusting for confounding factors, including physical function and PCS-12. That is, a higher CAVI value was associated with better HRQL in the physical dimensions and in the PCS-12.

These discrepancies between the ABI and CAVI results could be explained by the different phases of vascular aging that are evaluated by these parameters. CAVI alteration begins in the early stages of vascular aging, in which only the functional component is affected and the structure might still be normal. However, the ABI principally evaluates the vascular structure that affects more late phases. This could provide a possible explanation for the observation that quality of life is affected by ABI deterioration, because it reflects a more advanced stage of the atherosclerotic process. CAVI principally evaluates vascular function, in particular early impairment that has not yet affected quality of life.

Few studies to date have analyzed the relationship between ABI with the quality of life. Nevertheless, Long et al. [9] found a greater association between HRQL and PAD symptoms than with ABI values. However, Korhonen et al. [8] found that the HRQL of individuals with asymptomatic or borderline PAD was worse than in the subjects with normal ABI. Likewise, they concluded that the level of ABI is independently related to physical functioning. In the same line, Chen et al. [10] conducted a cross-sectional study with chronic hemodialysis patients and found that quality of life scores were positively and linearly associated with ABI values in patients with an ABI score below 0.9. This trend was also linear with an increase of the slope in patients with an ABI between 0.9 and 1.3. However, it became a negative for patients with an ABI score greater than 1.3. They concluded that ABI is not only an indicator of PAD but is also positively associated with quality of life [10]. These results are consistent with our findings.

Therefore, this study offers additional evidence for the relationship between ABI and quality of life in a large group of subjects with intermediate cardiovascular risk. We have no clear interpretation for the negative association, albeit at the limit of statistical significance, between ABI and the mental component of quality of life, and we cannot exclude it is a spurious association.

The association of vascular function with the quality of life has been less studied. Kidher et al. [39] found an association of the PWV with the quality of life in patients with severe aortic valve disease. Brunner et al. [19] found a negative correlation of carotid femoral PWV (cf-PWV) with the physical component of quality of life, as assessed by the SF-36 in an UK cohort. In our study, we only found a positive association of ba-PWV with physical and mental health; however, this relationship disappears when the model is adjusted for age and sex. The different methodologies used to calculate the PWV can influence the results between different studies.

Crilly et al. [20] studied rheumatoid arthritis patients and analyzed the relationship between quality of life (as measured by the Stanford Health Assessment Questionnaire disability index [40]) and AIx_75, and found a positive association between the two indexes that remained even after adjusting for potential confounders. The direction of the relationship of AIx_75 with the quality of life in our work in a population with intermediate cardiovascular risk is the same as that found by Crilly, although the association lost significance after adjusting for potential confounders. This difference might be attributable to the differences in study populations between the two studies.

We found an association between CAVI and the following dimensions: physical function, role physical, bodily pain, general health, mental health, and the PCS-12. After adjustment, the association only remained for the physical function dimension and the PCS-12. It is not clear why we noted a relationship between vascular function (as evaluated by CAVI) and quality of life (as assessed by the SF-12). Quality of life was found to be higher in men along most dimensions; age had a positive correlation with CAVI and some dimensions of quality of life, especially the mental component. The association of age and gender with quality of life and CAVI could be influencing the relationship between these two variables, but as shown in the second regression model, the associations persisted after adjusting for the above-mentioned variables.

Other possible variables that could mediate the relationship, such as lifestyle health behaviors, cardiovascular risk factors, or certain drugs, were included in the adjusted regression model, yet the association between CAVI and physical function remained even after adjusting for these potential mediators. When we conducted a literature review we did not find studies analyzing this relationship, and thus further studies are needed to confirm these findings.

### Limitations

The main limitation of this study was the use of cross-sectional data, which prevented us from establishing a temporal relationship between different parameters that assess vascular structure and function and HRQL. We must also bear in mind that the quality of life questionnaires have a subjective component that can influence the results. However, this is the first study to examine the relationship between ABI, ba-PWV, AIx_75, and CAVI with HRQL in adults with intermediate cardiovascular risk and normal ABI.

## Conclusions

For patients with ABI in the normal range, we found a positive association between the physical function dimension and the PCS-12 of HRQL; i.e., a higher ABI is associated with improved quality of life. The CAVI data also showed a positive association with the PCS-12, indicating that a more unfavorable CAVI was associated with improved quality of life. More studies are needed to clarify these findings and to assess whether vascular structure and function have different relationships with quality of life, or whether the results may be influenced by the subjective nature of the SF-12.

### Ethical approval and consent

The study was approved by an independent ethics committee from the Salamanca health area (Spain), and all participants gave written informed consent according to the general recommendations of the Helsinki Declaration.

### Consent for publication

Not applicable.

### Availability of data and materials

All relevant data supporting the conclusions of this article is included within the article.

## Additional files

Additional file 1: Table S1. Explanation of how MCS-12 and PCS-12 were calculated. (PDF 367 kb) Additional file 2: Table S2. Multiple linear regression analysis of vascular structure and function parameters and health-related quality of life. (PDF 383 kb)

## Abbreviations

ABI: ankle-brachial index
AIx: augmentation index
ba-PWV: brachial-ankle pulse wave velocity
BMI: body mass index
BP: Blood pressure
CAVI: cardio-ankle vascular index
cSBP: central systolic blood pressure
DBP: diastolic blood pressure
DQI: diet quality index
GLM: Multivariate General Linear Model
HAQ-DI: Stanford disability index
HbA1c: hemoglobin A1c
HDL: high-density lipoprotein
HRQL: health-related quality of life
IQR: interquartile range
LDL: low-density lipoprotein
LSD: Least significance difference.
LTPA: leisure time physical activity
MANOVA: multivariate analysis of variance
MAP: mean arterial pressure
MCS-12: mental component summary
PA: physical activity
PAD: peripheral arterial disease
PCS-12: physical component summary
pSBP: peripheral systolic blood pressure
SBP: systolic blood pressure
