# Supplementary material for: Vascular structure and function and their relationship with health-related quality of life in the MARK study
Source: BMC Cardiovasc Disord. 2016 May 12;16:95. doi: 10.1186/s12872-016-0272-9 (PMC4865998; doi:10.1186/s12872-016-0272-9)
Supplement: Additional file 1: Table S1. — Explanation of how MCS-12 and PCS-12 were calculated. (PDF 367 kb) [file 12872_2016_272_MOESM1_ESM.pdf]

Physical component summary (PCS-12) and Mental component summary (MCS-12).

The calculation of physical and mental component was carried out as detailed below:

Step 1:
COMPUTE AGG_PHYS = (PF_Z * 0.42402) + (RP_Z * 0.35119) + (BP_Z * 0.31754) + (GH_Z * 0.24954)+ (VT_Z * 0.02877) + (SF_Z * -0.00753) +(RE_Z * -0.19206) + (MH_Z * -0.22069).

COMPUTE AGG_MENT = (PF_Z * -0.22999) + (RP_Z * -0.12329) + (BP_Z * -0.09731) + (GH_Z * -0.01571) + (VT_Z * 0.23534) + (SF_Z * 0.26876) +(RE_Z * 0.43407) + (MH_Z * 0.48581).

Step 2:
COMPUTE PCS_US = 50 + (AGG_PHYS * 10).
COMPUTE MCS_US = 50 + (AGG_MENT * 10).
