# Supplementary material for: Vascular structure and function and their relationship with health-related quality of life in the MARK study
Source: BMC Cardiovasc Disord. 2016 May 12;16:95. doi: 10.1186/s12872-016-0272-9 (PMC4865998; doi:10.1186/s12872-016-0272-9)
Supplement: Additional file 2: Table S2. — Multiple linear regression analysis of vascular structure and function parameters and health-related quality of life. (PDF 383 kb) [file 12872_2016_272_MOESM2_ESM.pdf]

Table S2: Multiple linear regression analysis of vascular structure and function parameters and health-related quality of life.

a)	Model 1: Unadjusted				Model 2: Minimally adjusted				Model 3: Fully adjusted			
	B	CI95%		p	B	CI95%		p	B	CI95%		p
**ABI**												
STANDARDIZED PHYSICAL FUNCTION	29.42	16.11	42.73	<0.001	25.61	11.70	39.51	<0.001	26.22	11.68	40.75	<0.001
STANDARDIZED ROLE PHYSICAL	15.92	1.99	29.85	0.025	9.55	-4.92	24.02	0.195	10.60	-4.48	25.69	0.167
STANDARDIZED BODILY PAIN	21.82	4.18	39.46	0.015	8.05	-9.79	25.89	0.375	11.25	-7.99	30.49	0.251
STANDARDIZED GENERAL HEALTH	12.98	-0.63	26.60	0.061	9.76	-4.45	23.98	0.178	8.37	-6.59	23.32	0.272
STANDARDIZED VITALITY	13.29	-3.76	30.34	0.126	9.08	-8.81	26.96	0.319	6.60	-12.02	25.22	0.486
STANDARDIZED SOCIAL FUNCTIONING	1.02	-13.93	15.97	0.893	-7.87	-23.16	7.43	0.312	-12.94	-29.26	3.37	0.120
STANDARDIZED ROLE EMOTIONAL	4.72	-10.16	19.59	0.533	-3.49	-18.83	11.85	0.655	-8.32	-24.71	8.06	0.318
STANDARDIZED MENTAL HEALTH	14.69	-1.26	30.63	0.071	1.79	-14.21	17.79	0.826	-6.85	-23.60	9.90	0.422
**STANDARDIZED PHYSICAL COMPON.**	24.46	10.40	38.52	0.001	19.80	5.09	34.51	0.009	23.90	8.24	39.55	0.003
**STANDARDIZED MENTAL COMPONENT**	1.53	-14.55	17.61	0.852	-8.63	-25.02	7.77	0.301	-17.43	-34.67	-0.19	0.048
**CAVI**												
STANDARDIZED PHYSICAL FUNCTION	1.34	0.42	2.26	0.004	1.97	0.87	3.08	<0.001	1.96	0.79	3.13	0.001
STANDARDIZED ROLE PHYSICAL	1.51	0.57	2.45	0.002	1.39	0.26	2.53	0.017	1.05	-0.16	2.26	0.087
STANDARDIZED BODILY PAIN	2.02	0.83	3.21	0.001	1.40	0.00	2.81	0.050	1.11	-0.43	2.65	0.158
STANDARDIZED GENERAL HEALTH	1.00	0.07	1.92	0.035	0.51	-0.62	1.63	0.378	0.50	-0.71	1.70	0.416
STANDARDIZED VITALITY	0.56	-0.60	1.73	0.341	0.17	-1.25	1.58	0.818	-0.58	-2.07	0.92	0.449
STANDARDIZED SOCIAL FUNCTIONING	1.01	0.00	2.02	0.051	0.01	-1.20	1.22	0.984	-0.17	-1.49	1.14	0.798
STANDARDIZED ROLE EMOTIONAL	0.81	-0.20	1.82	0.115	0.14	-1.07	1.35	0.822	-0.15	-1.47	1.17	0.826
STANDARDIZED MENTAL HEALTH	1.57	0.49	2.64	0.004	0.49	-0.78	1.75	0.451	0.46	-0.89	1.80	0.504
**STANDARDIZED PHYSICAL COMPON.**	1.50	0.54	2.46	0.002	1.77	0.61	2.93	0.003	1.59	0.32	2.85	0.014
**STANDARDIZED MENTAL COMPONENT**	0.81	-0.28	1.90	0.145	-0.43	-1.73	0.87	0.513	-0.72	-2.11	0.67	0.310

b)	Model 1: Unadjusted			Model 2: Minimally adjusted			Model 3: Fully adjusted		
	B	CI95%	p	B	CI95%	p	B	CI95%	p
**ba-PWV**									
STANDARDIZED PHYSICAL FUNCTION	0.12	-0.28 0.52	0.549	0.24	-0.20 0.69	0.277	0.15	-0.34 0.64	0.542
STANDARDIZED ROLE PHYSICAL	0.49	0.09 0.89	0.017	0.46	0.01 0.91	0.043	0.24	-0.26 0.74	0.337
STANDARDIZED BODILY PAIN	0.37	-0.14 0.89	0.153	0.18	-0.37 0.74	0.515	-0.06	-0.70 0.58	0.850
STANDARDIZED GENERAL HEALTH	-0.02	-0.41 0.38	0.938	-0.28	-0.72 0.17	0.222	-0.33	-0.82 0.17	0.195
STANDARDIZED VITALITY	0.05	-0.45 0.54	0.853	-0.08	-0.64 0.48	0.777	-0.41	-1.02 0.21	0.195
STANDARDIZED SOCIAL FUNCTIONING	0.28	-0.15 0.71	0.197	0.00	-0.47 0.48	0.986	-0.06	-0.60 0.48	0.823
STANDARDIZED ROLE EMOTIONAL	0.19	-0.24 0.62	0.374	0.02	-0.45 0.50	0.919	-0.07	-0.61 0.47	0.794
STANDARDIZED MENTAL HEALTH	0.49	0.03 0.95	0.036	0.23	-0.27 0.73	0.363	0.25	-0.31 0.80	0.381
**STANDARDIZED PHYSICAL COMPON.**	0.19	-0.22 0.61	0.361	0.20	-0.27 0.66	0.401	0.00	-0.53 0.52	0.993
**STANDARDIZED MENTAL COMPONENT**	0.29	-0.18 0.75	0.226	-0.02	-0.53 0.49	0.933	-0.08	-0.65 0.50	0.791
**AIx_75**									
STANDARDIZED PHYSICAL FUNCTION	-0.09	-0.16 -0.02	0.014	-0.06	-0.14 0.02	0.134	-0.08	-0.16 0.00	0.052
STANDARDIZED ROLE PHYSICAL	-0.04	-0.12 0.03	0.240	-0.03	-0.11 0.05	0.504	-0.04	-0.13 0.04	0.301
STANDARDIZED BODILY PAIN	-0.08	-0.17 0.01	0.099	-0.04	-0.13 0.06	0.446	-0.05	-0.16 0.06	0.341
STANDARDIZED GENERAL HEALTH	-0.02	-0.09 0.05	0.582	-0.03	-0.10 0.05	0.505	-0.03	-0.11 0.06	0.521
STANDARDIZED VITALITY	0.00	-0.09 0.08	0.924	0.01	-0.09 0.11	0.840	0.03	-0.07 0.13	0.590
STANDARDIZED SOCIAL FUNCTIONING	-0.01	-0.08 0.07	0.887	0.00	-0.08 0.09	0.943	0.01	-0.08 0.10	0.865
STANDARDIZED ROLE EMOTIONAL	-0.01	-0.09 0.06	0.722	0.01	-0.08 0.09	0.891	0.01	-0.08 0.10	0.890
STANDARDIZED MENTAL HEALTH	-0.01	-0.09 0.07	0.834	0.02	-0.06 0.11	0.575	0.05	-0.04 0.14	0.293
**STANDARDIZED PHYSICAL COMPON.**	-0.08	-0.15 0.00	0.039	-0.06	-0.14 0.02	0.151	-0.08	-0.17 0.00	0.059
**STANDARDIZED MENTAL COMPONENT**	0.02	-0.06 0.11	0.625	0.04	-0.05 0.13	0.395	0.06	-0.03 0.16	0.183

Multiple linear regression analysis by Multivariate General Lineal model (GLM). Dependent variables: standardized physical function, standardized role physical, standardized bodily pain, standardized general health, standardized vitality, standardized social functioning, standardized role emotional, standardized mental health and Standardized Physical and Mental Component. Independent Variables: Ankle brachial index (ABI), Cardio Ankle Vascular index (CAVI), Brachial-Ankle Pulse Wave Velocity (ba-PWV) and Augmentation index (AIx_75).

Model 1: Unadjusted; Model 2: Adjusted for age and gender; Model 3: Adjusted for age, gender, current smoker, alcohol consumption in gr/week, physical exercise (METs-min 14 days), diet quality index, mean blood pressure, atherogenic index (CT/HDL-C), HbA1c and antihypertensive and lipid-lowering drugs.
